# EASIUR-HR: A Model To Evaluate Exposure Inequality Caused by Ground-Level Sources of Primary Fine Particulate Matter

**DOI:** 10.1021/acs.est.2c06317

**Published:** 2023-02-21

**Authors:** Brian M. Gentry, Allen L. Robinson, Peter J. Adams

**Affiliations:** † Department of Mechanical Engineering, Carnegie Mellon University, 5000 Forbes Avenue, Pittsburgh, Pennsylvania 15213, United States; ‡ Department of Engineering and Public Policy, Carnegie Mellon University, 5000 Forbes Avenue, Pittsburgh, Pennsylvania 15213, United States; § Carnegie Mellon University Africa, BP 6150 Kigali, Rwanda

**Keywords:** environmental justice, exposure disparity, PM_2.5_ exposure, reduced-complexity model, high resolution, vehicle electrification

## Abstract

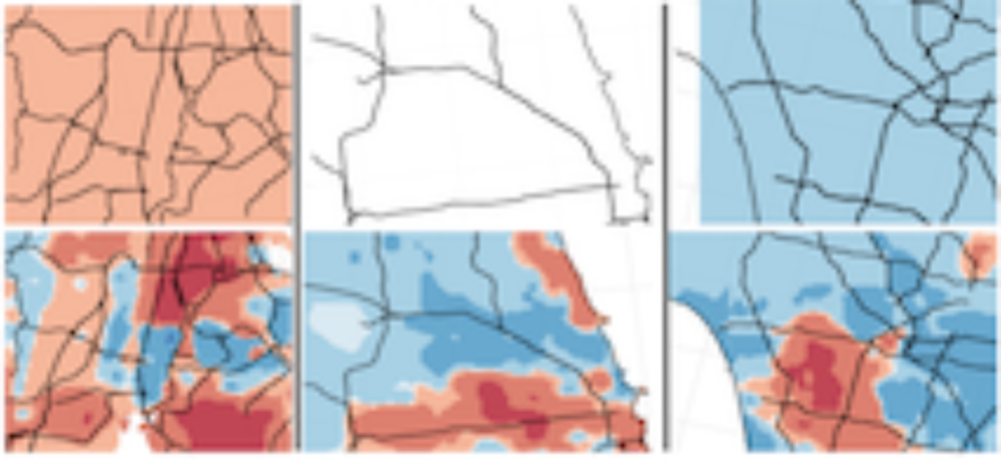

People of color disproportionately bear the health impacts of air pollution, making air quality a critical environmental justice issue. However, quantitative analysis of the disproportionate impacts of emissions is rarely done due to a lack of suitable models. Our work develops a high-resolution reduced-complexity model (EASIUR-HR) to evaluate the disproportionate impacts of ground-level primary PM_2.5_ emissions. Our approach combines a Gaussian plume model for near-source impacts of primary PM_2.5_ with a previously developed reduced-complexity model, EASIUR, to predict primary PM_2.5_ concentrations at a spatial resolution of 300 m across the contiguous United States. We find that low-resolution models underpredict important local spatial variation of air pollution exposure to primary PM_2.5_ emissions, potentially underestimating the contribution of these emissions to national inequality in PM_2.5_ exposure by more than a factor of 2. We apply EASIUR-HR to analyze the impacts of vehicle electrification on exposure disparities. While such a policy has small aggregate air quality impacts nationally, it reduces exposure disparity for race/ethnic minorities. Our high-resolution RCM for primary PM_2.5_ emissions (EASIUR-HR) is a new, publicly available tool to assess inequality in air pollution exposure across the United States.

## Abstract 2

This work describes a model to predict mortality and impacts of primary PM_2.5_ emissions by race/ethnicity to quantitatively evaluate environmental injustice caused by these emissions.

## 1 Introduction

Despite major improvements in domestic air quality over the last few decades, air pollution remains a critical environmental justice (EJ) issue.^2,12^ On a national scale, the most damaging air pollutant is particulate matter with diameter less than 2.5 μm, or PM_2.5_. When inhaled, PM_2.5_ penetrates deep into the lungs and can enter the bloodstream, increasing the risk of premature death from ischemic heart disease and other pulmonary and cardiac disorders.^21,22^ Nationally, exposure to PM_2.5_ causes between 100 000 and 200 000 premature deaths annually,^11,34^ a burden borne disproportionately by people of color.^23,34,35^ On average, communities of color experience PM_2.5_ concentrations 20% higher than white non-Hispanic/Latino communities.^34^

This increased health impact on communities of color follows a larger trend of documented environmental injustice in the United States. Beginning in the early 1980s, environmental justice researchers and activists described the disproportionate impact of environmental blights such as toxic waste sites and landfills on minority communities.^1,3^ More recent work has demonstrated a disproportionate impact on disadvantaged communities in other environmental realms, including exposure to PM_2.5_ and air toxics,^25,35^ water quality,^24^ and urban heat islands.^17^ Continued environmental impacts on disadvantaged communities elevate the need for policies to address environmental injustice.

Environmental justice has been a focus of federal decision makers for decades, since a standing executive order from the Clinton Administration requiring federal agencies to include an assessment of environmental justice in policy decisions. More recently, the Biden administration’s Justice40 initiative requires that 40% of benefits of some climate policy accrue to disadvantaged communities. To satisfy these federal requirements, policy makers need models to estimate a priori the potential of a policy to remedy environmental injustice.

Models need a high spatial resolution to resolve exposure disparities^28^ and ideally would be easy to use with limited computational burden to facility widespread adoption for policy analysis. In the field of air quality, the traditional approach to predicting the impacts of air pollution policies uses chemical transport models (CTMs) to estimate PM_2.5_ concentrations from a set of emissions. These models discretize an area of interest into grid cells, solving complex partial differential equations within each cell based on emissions, chemical reaction, and deposition in each cell, as well as transport between them. While CTMs remain the state-of-the-science models for simulating changes in PM_2.5_ concentrations from perturbed emissions inventories, they require substantial experience and computational resources to run. To address this challenge, researchers have developed reduced-complexity models (RCMs), which simplify the full simulation pathway and allow for predictions of both PM_2.5_ concentrations and the monetized health impacts (i.e., social costs) of PM_2.5_ concentrations from a perturbed set of emissions much faster than traditional models can.

A variety of RCMs have been developed.^9,14−16,26,36^ These models use different approaches, including regression models fit to results from CTM simulations^15,16^ and Gaussian plume frameworks^26^ to predict PM_2.5_ concentrations. Intercomparison between models has shown reasonable agreement between different RCMs,^10^ increasing confidence in ensemble results when multiple RCMs with different underlying assumptions are applied to the same policy. However, a shortcoming of most existing RCMs is low spatial resolution, e.g., 36 km^15^ or county level,^26^ a limitation which likely underestimates EJ impacts since it does not account for intraurban spatial variation in emissions profiles.^28^ A notable exception is InMAP, which provides predictions at 1 km resolution in some major urban areas. Developing additional RCMs to perform high-resolution analyses is critical for better understanding contributions toPM_2.5_ exposure disparity, as ensemble results are more robust than results from an individual RCM.^10^

This paper describes a new RCM, high-resolution EASIUR (EASIUR-HR), to predict contributions of ground-level primary PM_2.5_ emissions to PM_2.5_ exposure disparities by race at a high spatial resolution across the contiguous United States. This model builds upon EASIUR,^15^ a 36 km resolution RCM across the United States. EASIUR-HR predicts the social cost and disparate impacts of primary PM_2.5_ emissions at a 300 m resolution across the United States. To illustrate EASIUR-HR, we apply it to investigate the effects of vehicle electrification on PM_2.5_ exposure disparities. Past studies investigating vehicle electrification suggest it may present a net cost to society from an air quality perspective without a cleaner electric grid and little benefit to reduce PM_2.5_ exposure in disadvantaged communities.^18−20,33,40^ However, these studies used low-resolution models to evaluate inequity, which may underpredict spatial variation in pollution concentration and resulting exposure disparities.^28^

## 2 Materials and Methods

### 2.1 Model Development

Our goal in this paper is to develop an RCM to rapidly predict the change in primary PM_2.5_ concentration due to a marginal increase in ground-level primary PM_2.5_ emissions at a high enough spatial resolution to match the spatial variation in demographics (neighborhood level in urban areas), which will allow for predicting the disproportionate impact of emissions on race/ethnic groups. We built our RCM upon base EASIUR, an RCM developed at 36 km to predict the marginal change in PM_2.5_ concentration due to a marginal increase in emissions of primary PM_2.5_ and three PM_2.5_ precursors: NO_*x*_, SO_2_, and NH_3_. Base EASIUR is developed using a regression of the response of PM_2.5_ concentrations to changes in emissions based on a sample of 50 CTM runs; a full description of the methodology is given in ref (15). For this paper, we focused on increasing the spatial resolution for treatment of inert, primary PM_2.5_ only, as these emissions have the highest social costs per tonne of emissions^15,26,33^ and display the sharpest concentration gradients near the source. We do not provide high-resolution treatment of any other PM_2.5_ precursor present in the base EASIUR model.

To increase the spatial resolution of base EASIUR, we used AERMOD to generate concentration predictions at a resolution of 300 m within 72 km of a source location. These concentration predictions replace those from base EASIUR, which provides concentration predictions outside the local area. We repeated this approach for 94 million source locations across the contiguous United States, terming this modeling approach EASIUR-HR. This approach is similar to that used by the EPA in its National Air Toxics Assessment (NATA).^5^

Many past studies have evaluated AERMOD in a variety of environments, including complex terrain and urban environments. They found that AERMOD predicts maximum annual-average concentrations (at distances less than 1 km to the source) within 20–40%.^13,29,30^ Additionally, Gaussian plume models are derived directly from the conservation of mass, and substantial work since the 1950s has gone into developing accurate parametrization of the dispersion parameters used in the model,^32,38^ making AERMOD a robust model for predicting the high-resolution spatial variation in concentration.

**Figure 1:**
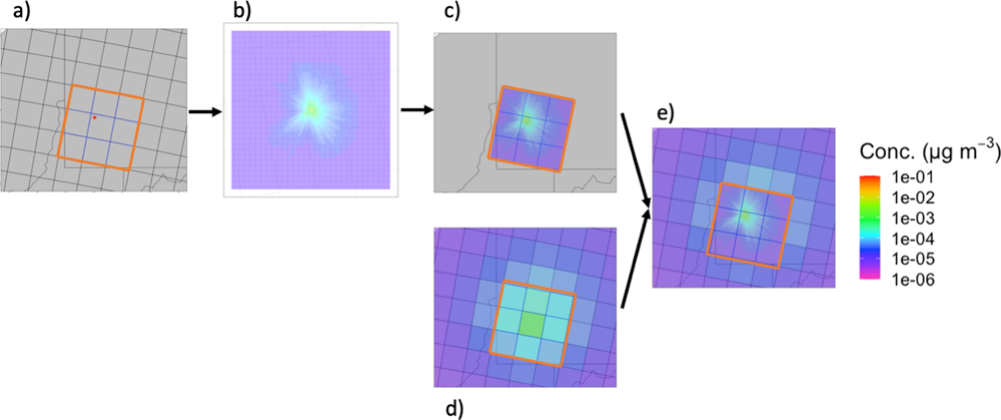
Flowchart of EASIUR-HR methodology. (a) Source location over downtown Pittsburgh, identifying the nine local base EASIUR grid cells. (b) AERMOD prediction of local PM_2.5_ concentrations from that source location. (c) Concentration predictions from b centered on the source location and cropped at the local cell boundary. (d) Base EASIUR concentration predictions on the base EASIUR grid. (e) Combined concentration field with AERMOD predictions replacing those from base EASIUR.

We illustrate EASIUR-HR in Figure 1. For a given source location, we identify the cells highlighted in blue (the nine base EASIUR cells centered on the base EASIUR cell in which the source is located) as the “local cells”. We run AERMOD to generate concentration predictions from the source within these nine local cells, while we use base EASIUR to predict the concentration outside the local cells. We repeat this process for all 94 million source locations, located on a 300 m grid, across the United States.

We made two simplifying assumptions in the development of EASIUR-HR. First, we used meteorology data from the nearest meteorological station in the ASOS program rather than on-site meteorological data, assigning each source location to an entire “meteorology region”, an area that uses the same meteorology data. In total, we had 517 meteorology regions (and corresponding 517 sets of meteorology data); a map of meteorology regions can be found in Figure S16. Second, we neglected terrain effects, assuming flat terrain. Both of these assumptions are also made by the EPA in its development of the NATA.^5^ Making these two assumptions allowed us to generate a set of average plumes; if meteorology and terrain inputs are the same, a Gaussian plume model will predict the same concentration fields, and hence, two sources in the same meteorology region have identical concentration fields, just translated in space. We evaluated the error introduced by these two assumptions empirically, as described below.

We used the 300 m resolution PM_2.5_ concentration predictions to evaluate two exposure metrics: (1) social cost due to premature mortality and (2) differential exposure across race/ethnic groups. For 1, we used the Cox hazards model described in eq 1 to estimate a change in premature mortality, Δ*m*, resulting from a change in PM_2.5_ concentration, *C_j_*.
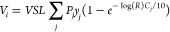
1In this equation, *P* is the population in a grid cell, *y* is the baseline mortality rate in a grid cell, and *R* is the risk ratio (here assumed to be 1.06^21^). The sum is taken over all grid cells across the contiguous United States. We monetized Δ*m* using a VSL of $8.6 million (2010 $USD)^15,39^ to obtain social cost estimates.

To predict differential exposure across race/ethnic groups, we define a source-oriented disparity metric on demographic group *k* from emissions in source cell *i*, *D*_*k*,*i*_ (in μg·m^–3^·t^–1^), defined by eq 2, representing the change in exposure disparity across the entire contiguous United States due to a change in emissions in one 300 m grid cell.
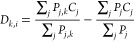
2In eq 2, *P*_*k*,*j*_ represents the population of race/ethnic group *k* in cell *j*, *P*_*j*_ is the total population in cell *j*, and *C*_*j*_ is the PM_2.5_ concentration in cell *j* from unit emissions from cell *i*.

We empirically evaluated the error introduced by the two assumptions: meteorology and terrain. To evaluate our meteorology assumption, we substituted the meteorology used at 18 metropolitan areas in the United States with meteorological data from a different ASOS station. We then recalculated source-oriented social costs and disparity metrics at each of the EASIUR-HR grid cells and calculated the mean absolute error (MAE) between the two estimates. We estimated the conditional mean of these MAE values with emissions values, as a nonzero conditional mean would introduce bias into EASIUR-HR estimates. To evaluate our terrain assumption, we replaced the AERMOD simulation run without including the effects of terrain with one including the effects of terrain at 1700 locations in a variety of terrain environments. We then recalculated source-oriented social costs and disparity metrics, estimating the MAE between the simulation without and with the effects of terrain included.

### 2.2 Case Study: Vehicle Electrification

To illustrate the application of EASIUR-HR, we examined the distribution of air quality impacts of passenger vehicle electrification across race/ethnic groups. Passenger vehicles emit substantial amounts of primary PM_2.5_ and NO_*x*_ in vehicle exhaust, emissions that are eliminated if gasoline vehicles are replaced by electric vehicles. However, this switch will increase electricity demand, potentially increasing emissions of primary PM_2.5_, NO_*x*_, and SO_2_ depending on the mix of sources for electricity generation. We modeled both of these effects using EASIUR-HR to predict changes in concentrations of passenger vehicle exhaust primary PM_2.5_ and base EASIUR to predict changes in PM_2.5_ concentrations due to changes in vehicle exhaust NO_*x*_ and all electricity generation emissions. We present two bounding cases for national vehicle electrification: vehicle electrification under the current electricity grid (EV-CUR) using emissions factors from 2018 power plant data and vehicle electrification under an all-renewable grid (EV-REN), assuming no air pollutant emissions from electricity generation.

We use the EPA’s MOtor Vehicle Emission Simulator (MOVES)^8^ to generate county-level vehicle exhaust emissions of primary PM_2.5_ and NO_*x*_ from 2019 and then allocated them to the base EASIUR grid using a population-weighted average. After estimating emissions on the base EASIUR grid, we allocated the emissions in each base EASIUR grid cell to the 300 m grid EASIUR-HR operates on using spatial surrogates. MOVES automotive emissions are classified by three road types: off-network, unrestricted access roads, and restricted access roads. We used population density as a surrogate to assign off-network emissions to the 300 m grid and road length by road type as a surrogate for unrestricted and restricted access roads using 2018 road network data from OpenStreetMap.^27^ We classify motorways, trunk roads, and primary roads as restricted access roads and all other roads as unrestricted access. We allocate the grid cell emissions by road type to the 300 m EASIUR-HR grid and then add up contributions from each road type to get a total estimate of vehicle exhaust primary PM_2.5_ emissions at a 300 m resolution across the country.

To estimate changes in power plant (electricity generating unit, or EGU) air pollutant emissions to meet the demands of electric vehicles under EV-CUR, we follow the methodology of Holland et al.,^19^ estimating emissions factors econometrically. We regress hourly SO_2_, NO_*x*_, and PM_2.5_ emissions at the plant level against hourly electricity demand within each North American Electric Reliability Corporation (NERC) region in the same interconnect as the plant using emissions and load from the year 2019.^4,6,7^ Unlike Holland et al., we do not include hour-of-day effects in our regression; this assumption precludes the use of nonconstant vehicle charging profiles. The regression coefficients of emissions against regional load provide marginal emissions factors, which we then apply to an annual estimated increase in load due to electric vehicle charging, proportional to total VMT using an estimated vehicle efficiency of 100 MPGe. For EV-REN, we assume that there were no additional emissions associated with producing the electricity for electric vehicles. We also performed sensitivity analyses to dispatch model^31^ and our assumed vehicle efficiency.

## 3 Results and Discussion

**Figure 2:**
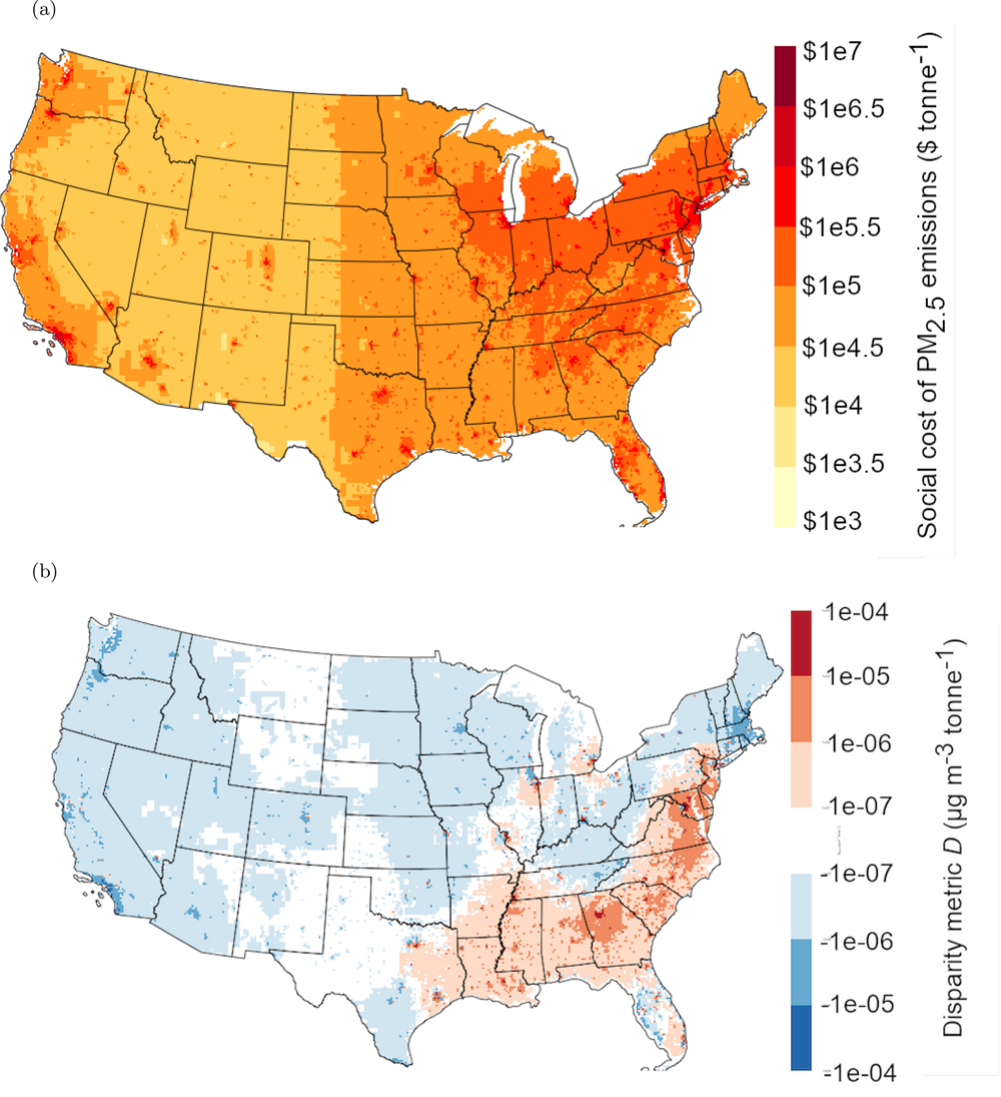
(a) EASIUR-HR predicted social cost, and (b) disparity metric for Black vs total predictions plotted across the contiguous United States at 300 m resolution.

We first investigated the changes in predicted social cost and exposure disparity when increasing grid resolution from 36 km to 300 m. For social cost, increasing grid resolution produces additional spatial variation in source-oriented social costs compared to base EASIUR (Figure 2a) but with largely similar regional trends to base EASIUR. Social costs of emissions are higher in more densely populated areas compared to rural areas, as indicated by darker shades of red, reaching a maximum of $12 million tonne^–1^ for emissions in New York City. This follows the trend of base EASIUR, which predicts higher social costs in major urban areas.

Within urban areas, EASIUR-HR predicts social cost variation that is much larger than that predicted by base EASIUR. For example, in Los Angeles County (Figure S15), EASIUR-HR predicts social costs ranging from $50 000 in the rural San Gabriel Mountain Range north of the city to more than $4.5 million in the middle of downtown. In contrast, base EASIUR predicts variation of the social cost from around $100 000 tonne^–1^ of primary PM_2.5_ emitted to around $500 000 tonne^–1^ closer to the urban core of Los Angeles, a variation by a factor of 5 compared to a factor of 90 in EASIUR-HR. However, while individual locations may have higher social costs than predicted in base EASIUR, the county-level average social costs only vary by 10% between base EASIUR and EASIUR-HR. EASIUR-HR is distributing the social costs of air pollution at a finer scale but not substantially changing the aggregate cost.

Our novel disparity metric (eq 2) shows a more varied spatial pattern than social cost. Figure 2b depicts *D*_Black,*i*_, the per tonne contribution to national exposure disparity between Black communities and the population as a whole across the contiguous United States. This metric accounts for the impacts across the entire country due to emissions from a single source location. This disparity metric attains values between −1.5 × 10^–4^ and 5 × 10^–4^ μg·m^–3^·t^–1^. The values here are small in magnitude because they represent the contribution of one tonne of primary PM_2.5_ emissions from a single location to national population-weighted average exposure disparity. Across much of the United States, the value of our disparity metric for Black communities against total population is essentially zero (areas in light blue, white, and light red), indicating that primary PM_2.5_ emissions from these locations do not substantially contribute to unequal PM_2.5_ exposure for Black communities at the national level. Across much of the South and into the mid-Atlantic states as well as in some major urban areas in the Midwest, the disparity metric achieves positive values (regions in dark red), indicating primary PM_2.5_ emissions disproportionately impact Black communities. In urban areas with relatively small Black populations, such as Seattle, the disparity metric achieves negative values (regions in dark blue), indicating primary PM_2.5_ emissions *reduce* unequal exposure for Black communities since these emissions have a disproportionate impact on other race/ethnic groups. These trends largely reflect the distribution of Black populations throughout the United States.

**Figure 3:**
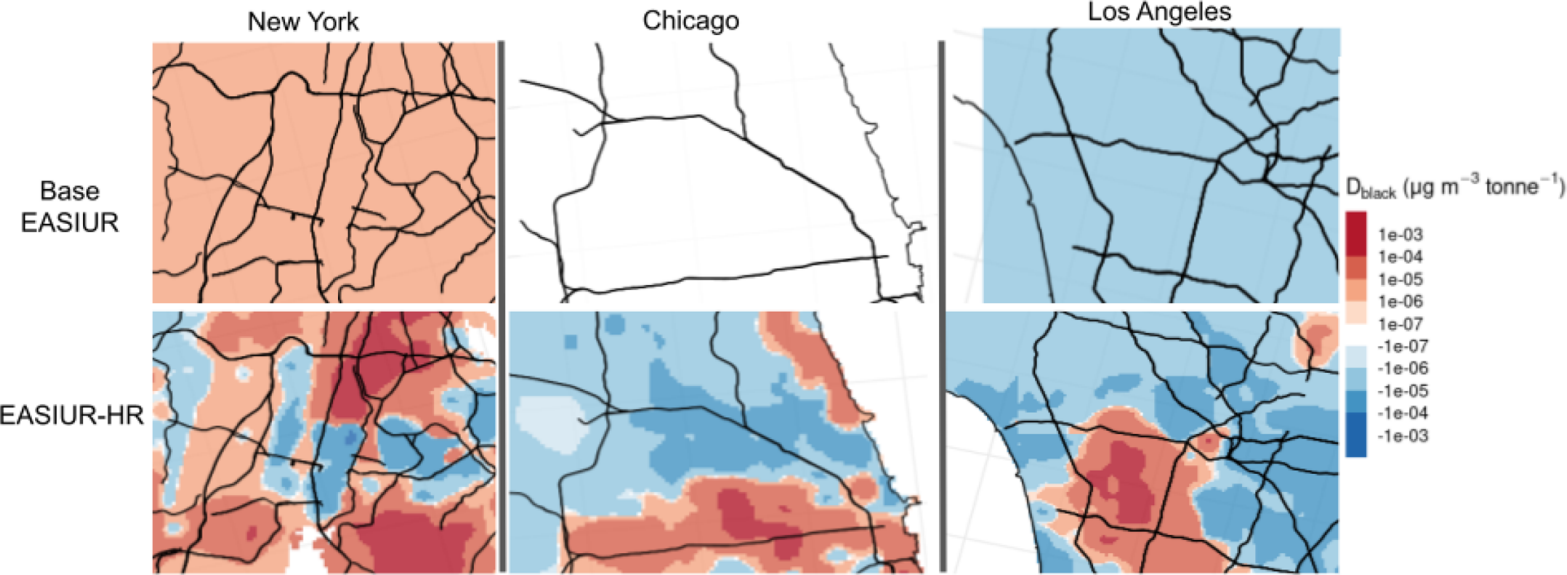
Disparity metric for Black populations plotted in New York, Chicago, and Los Angeles using base EASIUR and EASIUR-HR. EASIUR-HR reveals substantial intraurban variability in the disparity metric not seen in base EASIUR.

EASIUR-HR predicts much more intraurban variability in the disparity metric than base EASIUR. Figure 3 shows *D*_black_ plotted in three urban areas (New York City, Chicago, and Los Angeles) using predictions from base EASIUR and EASIUR-HR on the top and bottom, respectively. With a 36 km resolution, base EASIUR indicates that primary PM_2.5_ emissions in New York City will unilaterally increase disparity for Black communities, emissions in Chicago will have almost no impact on disparity for Black communities, and emissions in Los Angeles will decrease disparity for Black communities. In contrast, EASIUR-HR predicts substantial intraurban variability, with pockets of large magnitude disparity metric values (both positive and negative) in all three urban areas, indicating that emissions in these urban areas could disproportionately impact Black populations depending on where the emissions are located.

The substantial intraurban variability in social cost and disparity metric predicted by EASIUR-HR at 300 m resolution has major implications for interpreting results of models with low spatial resolution treatment of ground-level primary PM_2.5_. For example, without accounting for high spatial variability in emissions, a low-resolution (i.e., 36 km or county level) model would predict a *reduction* in inequality for Black communities as a result of an increase in emissions in Los Angeles County (Figure S16). However, EASIUR-HR predicts that inequality would increase due to emissions in many parts of Los Angeles (Figure 3). This variability highlights the importance of model resolution on social cost and equity analyses; if emissions profiles are spatially correlated with positive/negative disparity metric values, a low-resolution model may substantially over/underpredict the overall contribution of those emissions to social cost or national inequity.^28^

### 3.1 Effects of Spatial Resolution

To determine if 300 m resolution captures the full contribution of emissions of PM_2.5_ to exposure disparity, we investigated the variation of EASIUR-HR results with grid resolution. We considered a scenario where emissions are correlated with population density, a commonly used spatial surrogate, investigating the effect of model resolution on predicted contributions to disparity. We calculated a population-weighted *D*_*k*,*i*_ metric within a single base EASIUR grid cell in a major urban area (New York City) as model resolution was increased from 36 km to 300 m. We calculated the change in this metric for *k* ∈ {Black, Asian, Hispanic/Latino, white non-Hispanic/Latino}.

**Figure 4:**
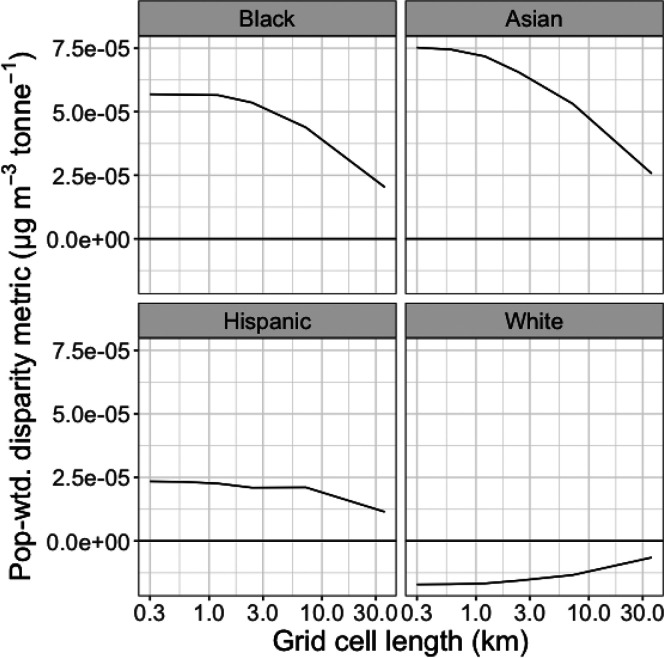
Variation of population-weighted national disparity metric in New York with grid resolution. Model converges below a grid cell length of 1 km for all race/ethnic groups modeled here, indicating that 300 m resolution is sufficient.

Model resolution has a significant impact on disparity metrics. For example, Figure 4 indicates that in New York City, increasing model resolution from 36 km to 300 m increases the population-weighted disparity metric by over a factor of 3 for each race/ethnic group. We find that our population-weighted equity metric is essentially constant below a grid cell length of 1 km (Figure 4), consistent with the results of Paolella et al.^28^ Since EASIUR-HR predictions are at 300 m (less than 1 km), they should capture the spatial variation in primary PM_2.5_ concentration required to match the spatial variation in demographics documented in the Census.

### 3.2 Evaluation of Assumptions and Uncertainty

We evaluated the potential uncertainty associated with our simplifying assumptions regarding meteorology and terrain effects.

#### 3.2.1 Meteorology Assumption

In our RCM, we simplified treatment of meteorology using the same meteorology data across “meteorology regions”. We evaluated the change in social cost and equity estimates associated with substituting meteorological data at 18 metropolitan areas across the country, defining the mean absolute error as the average absolute change in social cost/disparity metric when using meteorology data from the second-closest ASOS station. Across the 18 metro areas, substituting meteorology introduces a mean absolute error of $15 000 tonne^–1^ (9% of the average social cost value across the metro areas) in social cost estimates and a mean absolute error of 2.7 × 10^–7^ μg·m^–3^·t^–1^ (27% of the average disparity metric value across the metro areas) in disparity metric estimates from the base values used in the model. We also plotted EASIUR-HR residuals (i.e., the mean absolute error associated with our meteorology assumption) against two spatial emissions profiles, population-weighted and road length-weighted emissions profiles, to investigate correlations of EASIUR-HR errors with emissions, something that would introduce bias in aggregate estimates. EASIUR-HR errors have zero mean but display heavy tails, as shown in Q–Q plots (Figures S9 and S10). Our errors also appear to display zero conditional mean with emissions, with points generally falling equally above and below the *x* axis, as shown in Figures S11–S14. Since our residuals have zero conditional mean with emissions nationally, we can treat EASIUR-HR error associated with meteorology as random when analyzing emissions scenarios at the national scale. While the error can be significant at any one location, aggregate estimates of social cost and disparity due to emissions across the country are not biased by our meteorology assumption.

#### 3.2.2 Terrain Assumption

EASIUR-HR is based on AERMOD simulations that neglect the effects of local terrain. This dramatically reduces computational cost but introduces uncertainty. To evaluate the impact of neglecting terrain, we generated separate plumes at 1700 source grid cells within the same 18 urban areas, one including terrain for that location and one neglecting it, and recalculated social cost and equity metrics to evaluate bias and variability. We selected source grid cells in both flat and complex terrain environments. Neglecting terrain data biased social cost and equity metric estimates lowered by 10% relative to simulations including terrain and introduced a mean absolute error of $22 000 (13% of average value) in social cost estimates and 1.3 × 10^–7^ μg·m^–3^·t^–1^(15% of average value) in disparity metric estimates from the base values in the model. While errors associated with the neglect of terrain effects at a single location can be high, overall error when assessing national policies will be low for national policies with nationally distributed sources.

#### 3.2.3 Uncertainty Summary

In this section, we have assessed the uncertainty of our RCM relative to a full simulation with AERMOD. While social cost and equity metric values for an individual source location may vary by up to 30% due to meteorology and terrain assumptions, we can treat this variation as random, which disappears when averaging across a large number of sources distributed across the country. When our predictions are averaged across the 94 million EASIUR-HR grid cells, the ultimate uncertainty in national estimates that is specifically associated with simplified meteorology and terrain effects is very small.

### 3.3 Case Study: Vehicle Electrification

To illustrate EASIUR-HR, we applied it to estimate the impact of vehicle electrification of the U.S. passenger vehicle fleet on PM_2.5_ concentrations and PM_2.5_ exposure disparity. We assessed two national policy scenarios in which all vehicles are electrified: one in which the electricity required to power the electric vehicles is produced under the current power grid (EV-CUR), and one in which electricity to power the electric vehicles is produced using an all-renewable grid, i.e., one with no air pollutant emissions (EV-REN). These policies incorporate both the reduction in vehicle exhaust emissions of primary PM_2.5_ and NO_*x*_ and increased electric grid emissions of primary PM_2.5_, NO_*x*_, and SO_2_ under the current power grid scenario. We estimated PM_2.5_ concentrations from vehicle exhaust emissions of ground-level primary PM_2.5_ using EASIUR-HR and the remaining pollutants through base EASIUR, combining the resulting social cost and disparate impact estimates.

First, we consider the effect of electric vehicle adoption on overall air quality. For each policy scenario, we calculated the change in national population-weighted average PM_2.5_ concentration. EASIUR-HR predicts that the national population-weighted PM_2.5_ concentration under EV-CUR will negligibly increase by 0.01 μg·m^–3^, producing a social cost of $740 million (2010 $USD), or an additional 86 deaths per year. Using base EASIUR in place of EASIUR-HR yields an increase in PM_2.5_ concentration of just 0.001 μg·m^–3^, corresponding to a social cost of $80 million per year. These are both within the range of uncertainty for RCMs. Under EV-REN, PM_2.5_ concentrations would reduce substantially by 0.18 μg·m^–3^, producing a large social benefit of $14 billion per year.

**Figure 5:**
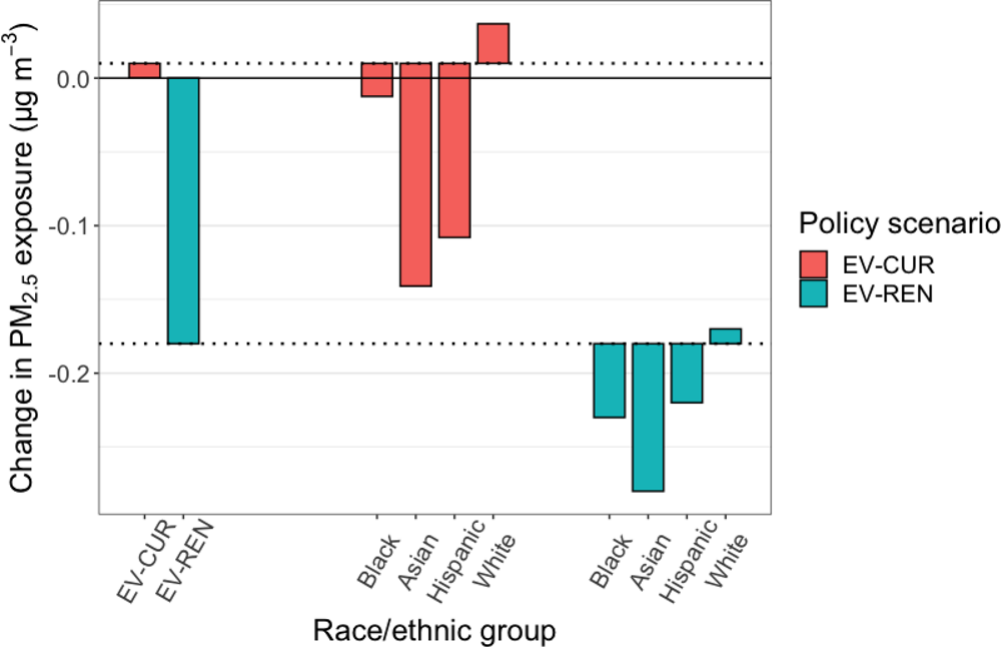
Modeled changes in race/ethnic PM_2.5_ exposure disparity for two policy scenarios, EV-CUR and EV-REN. Dashed lines indicate the overall changes in population-weighted PM_2.5_ concentrations under each policy, while bars indicate the additional change in PM_2.5_ concentration for each race/ethnic group plotted relative to the change in the national average. EASIUR-HR predicts almost no social benefit under EV-CUR but substantial benefit under EV-REN.

Although EV-CUR degrades current air quality, both EV-CUR and EV-REN reduce race/ethnic exposure disparities in PM_2.5_ exposure. Figure 5 shows the overall predicted change in population-weighted PM_2.5_ concentrations under both policy scenarios as well as the modeled changes in PM_2.5_ exposure disparity by race/ethnicity. Under both policy scenarios, we predict a larger population-weighted reduction in PM_2.5_ concentration for Black, Asian, and Hispanic/Latino communities than the population as a whole, reflecting the fact that these groups are disproportionately impacted by vehicle emissions and indicating that these policies will reduce PM_2.5_ exposure disparity. Under EV-REN, Black communities see a larger reduction in PM_2.5_ exposure disparity compared to the current grid while Asian and Hispanic/Latino communities see smaller reductions in disparity, reflecting the fact that Black communities are more impacted by EGU emissions than the U.S. population as a whole while Asian and Hispanic/Latino ones are not.^37^

Considering only vehicle exhaust primary PM_2.5_ emissions, EASIUR-HR predicts much higher changes in PM_2.5_ exposure disparity by race/ethnicity than base EASIUR for vehicle electrification. Figure S17 shows the predicted change in PM_2.5_ exposure disparity using the two models with the contribution of vehicle exhaust PM_2.5_ only. Here, bars below the dotted lines indicate that the race/ethnic group experiences a reduction in PM_2.5_ exposure compared to the population as a whole. With EASIUR-HR, we estimate a 2.5 times higher contribution of primary PM_2.5_ emissions compared to base EASIUR for white non-Hispanic/Latino, Black, and Asian communities and 7 times higher contribution for Hispanic/Latino communities. This is consistent with previous work,^28^ which showed that high spatial resolution is required to assess the equity implications of changes in PM_2.5_ emissions.

We examined the sensitivity of our results to a dispatch model, with details reported in the SI. We implemented the methodology detailed in Schnell et al.^31^ to allocate electricity demand to EGUs across the United States, finding that their methodology predicts higher power plant emissions of SO_2_, NO_*x*_, and PM_2.5_ but that overall exposure to resulting PM_2.5_ concentrations is lower, such that vehicle electrification reduces PM_2.5_ concentrations by 5 × 10^–3^ μg/m^3^ according to EASIUR-HR. The overall ranking of changes in disparity remains the same, with Asian and Hispanic/Latino communities seeing large reductions in PM_2.5_ concentration, followed by Black communities and finally white communities. Primary PM_2.5_ from vehicle exhaust remains a large contributor to overall inequity. Vehicle electrification, under both the current grid and a future all-renewable grid, produces substantial air quality benefit for communities of color, and these results are robust to dispatch model.

### 3.4 Discussion and Policy Implications

EASIUR-HR allows for a rapid assessment of the air quality benefits accrued to race/ethnic minority populations due to a change in primary PM_2.5_ emissions, something previously infeasible to do using conventional models or low-resolution RCMs. Our model results are publicly available upon request, aggregated to Census geographies from the block group through state resolution, for users to analyze their own policy scenarios. With a high-resolution emissions inventory of ground-level primary PM_2.5_ emissions, users can rapidly estimate the aggregate disparate impact of those emissions on race/ethnic groups across the country like they would with a social cost RCM, multiplying the emissions values (in tonnes) by the disparity metrics (in μg·m^–3^·t^–1^) and summing up the resultant products across the country. EASIUR-HR is suitable for analysis of national scenarios with a large number of sources, such as vehicle emissions as analyzed here or other nationally distributed sources. For an individual source or location, the uncertainties are larger. In these situations, more targeted models, such as AERMOD by itself, should be used.

EASIUR-HR neglects any atmospheric chemistry, ignoring high-resolution contributions to disparity caused by secondary PM_2.5_ precursors. For disparity caused by secondary PM_2.5_ precursors, we used base EASIUR at a 36 km resolution. This does not introduce error into our exposure disparity analysis because the atmospheric chemistry that creates secondary PM_2.5_ is not fast enough to create substantial new PM_2.5_ on a subkilometer scale. Secondary PM_2.5_ is important from a total PM_2.5_ perspective but has a more regional distribution rather than a local one, creating shallower spatial gradients due to the relatively slow atmospheric chemistry.

Our results from our electric vehicle policy scenario analysis indicate that aggregate air quality benefits from vehicle electrification are small without a substantial shift to renewable electricity. Despite small aggregate benefits, vehicle electrification under either EV-CUR or EV-REN reduces exposure disparity for Black, Asian, and Hispanic populations. EASIUR-HR predicts more than twice as much disparity caused by vehicle exhaust primary PM_2.5_ emissions than base EASIUR.

While here we have quantified the contribution of primary PM_2.5_ emissions to race/ethnic differences in population-weighted PM_2.5_ concentrations (one indicator of socioeconomic advantage), EASIUR-HR can easily be adapted to calculate benefits to government-classified disadvantaged communities. This allows for policy analysts to assess compliance, for example, with federal policy initiatives such as Justice40, a key initiative to address environmental injustice in areas such as air pollution exposure.

## Supporting Information Available

Figures of emissions inventories, sensitivity analysis, error analysis, intraurban variation in social cost and disparity metric values in Los Angeles County, meteorology regions, and changes in disparity associated with exhaust PM_2.5_ (PDF)
