# Supplementary material for: EASIUR-HR: A Model To Evaluate Exposure Inequality Caused by Ground-Level Sources of Primary Fine Particulate Matter
Source: Environ Sci Technol. 2023 Feb 21;57(9):3817–24. doi: 10.1021/acs.est.2c06317 (PMC9996819; doi:10.1021/acs.est.2c06317)
Supplement: Supplementary file 1 — es2c06317_si_001.pdf [file es2c06317_si_001.pdf]

EASIUR-HR: A MODEL TO EVALUATE EXPOSURE INEQUALITY CAUSED BY GROUND-LEVEL SOURCES OF PRIMARY FINE PARTICULATE MATTER – SUPPORTING INFORMATION

Brian M. Gentry[†‡*], Allen L. Robinson[†⋆], Peter J. Adams[‡]

[†]Carnegie Mellon University, Department of Mechanical Engineering, 5000 Forbes Ave, Pittsburgh, PA 15213

[‡]Carnegie Mellon University Department of Engineering and Public Policy, 5000 Forbes Ave, Pittsburgh, PA 15213

[⋆]Carnegie Mellon University Africa, BP 6150, Kigali, Rwanda

[*]Corresponding author

This supporting information file is 18 pages long and contains 17 figures.

1 Holland et al. emissions inventory

The following figures show the predicted changes in emissions of relevant precursor pollutants using Holland et al.'s methodology[1].

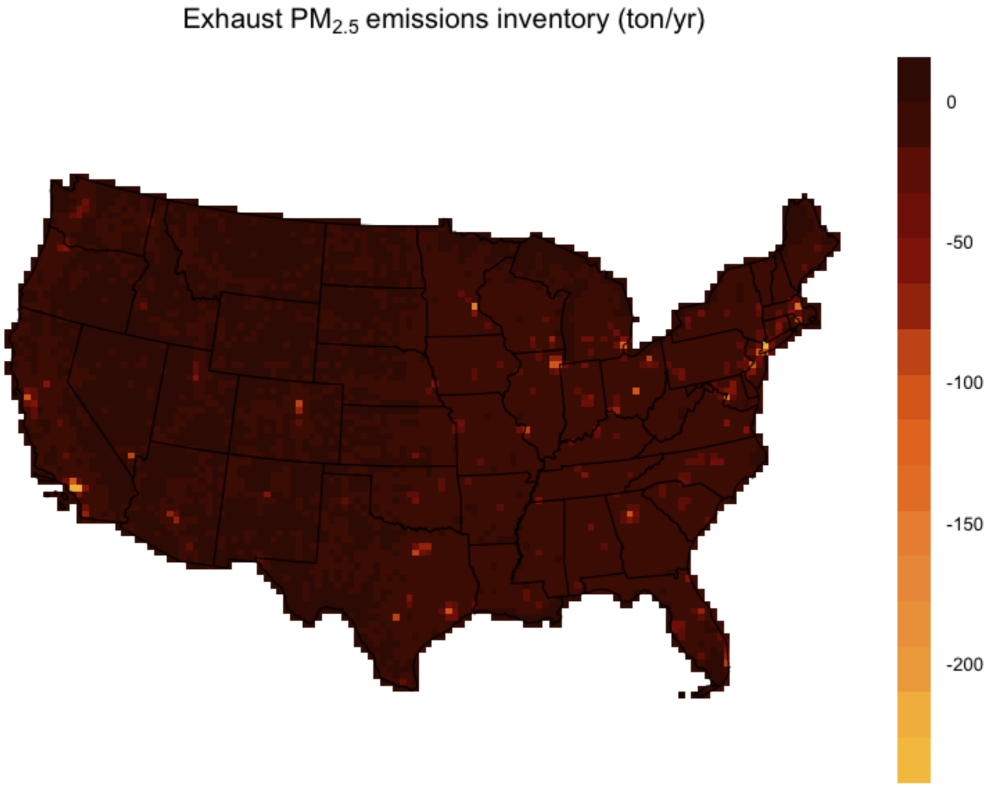

Figure S1: Exhaust $PM_{2.5}$ emissions inventory.

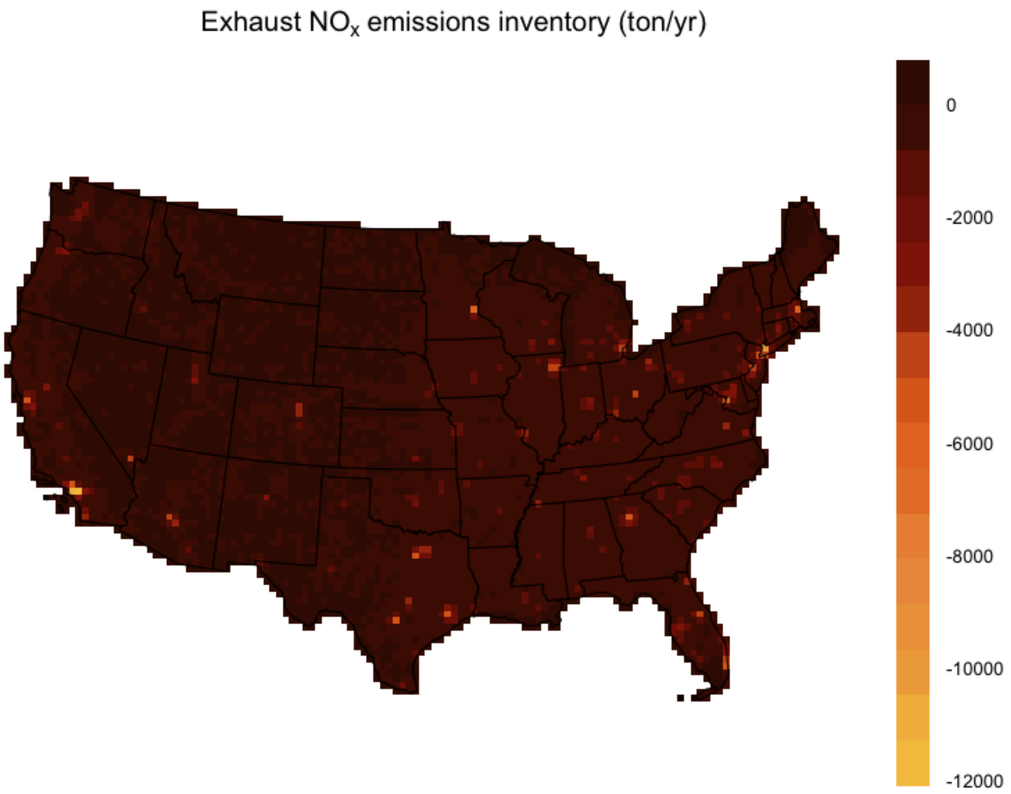

Figure S2: Exhaust NO_x emissions inventory.

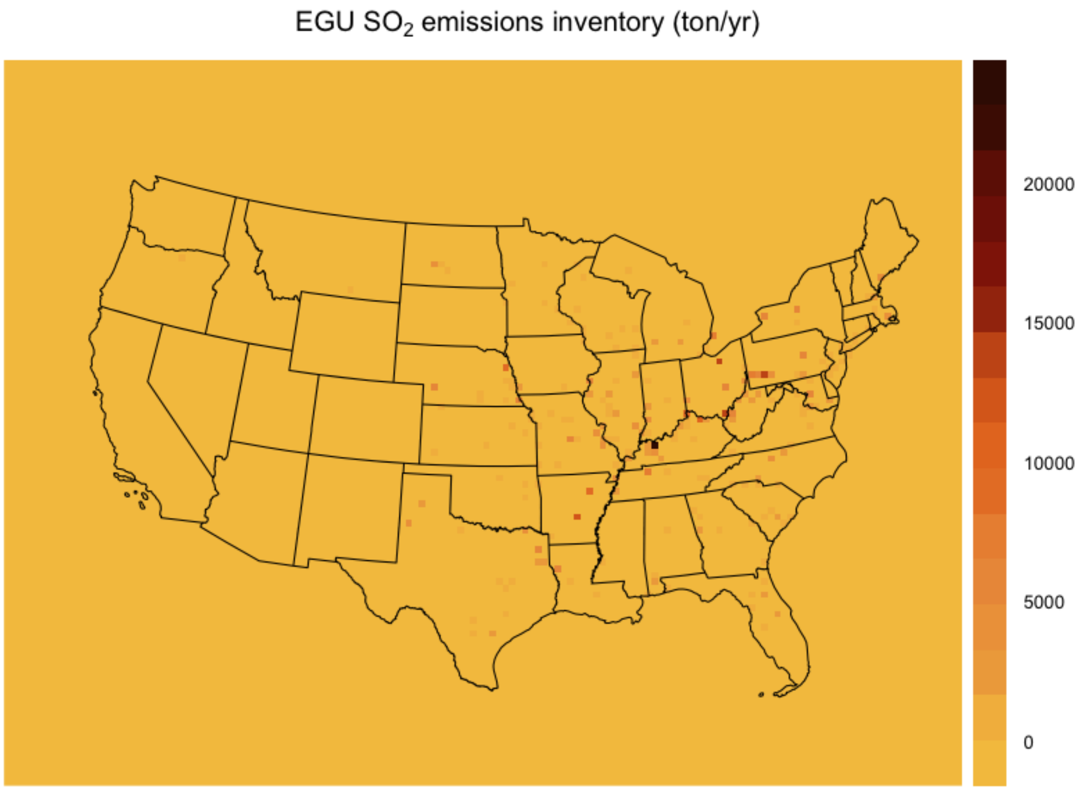

Figure S3: EGU SO_2 emissions inventory.

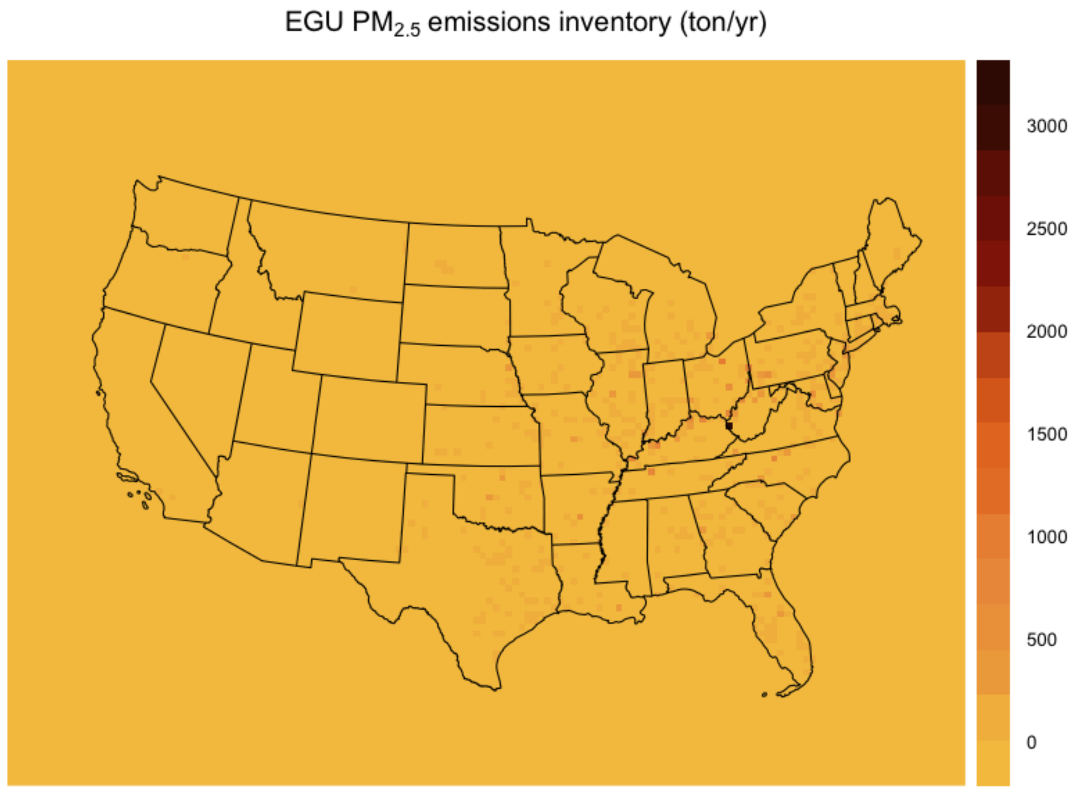

Figure S4: EGU $PM_{2.5}$ emissions inventory.

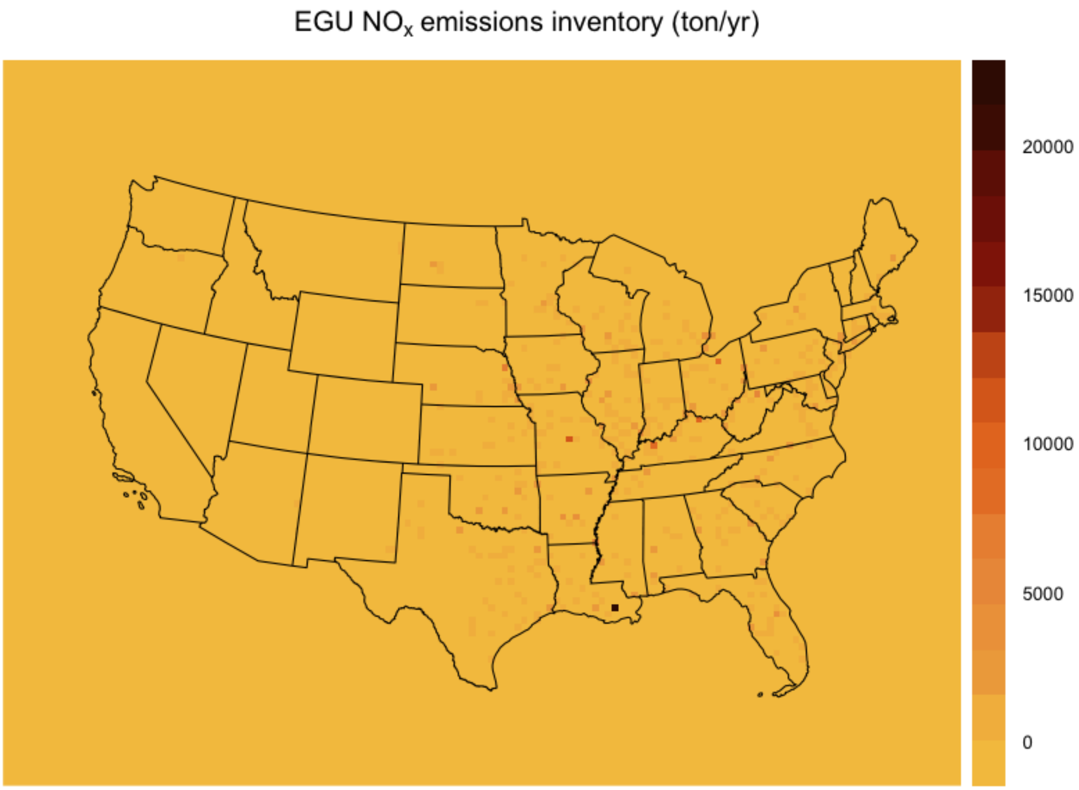

Figure S5: EGU NO$_x$ emissions inventory.

2 Holland et al. results

We processed the marginal change in emissions from Holland et al.'s methodology through the original, low-resolution EASIUR model; this plot is shown in Figures S6.

3 Schnell et al. results

We used Schnell et al.'s[2] methodology to estimate changes in EGU emissions. When processed through EASIUR-HR, our results suggest that vehicle electrification provides a net benefit to society, but only barely. If all passenger vehicles were electrified, population-weighted average $PM_{2.5}$ concentrations would decrease by an estimated 0.005 $\mu g/m^3$, corresponding to an annual social benefit of roughly $400 million (2010 $USD, $8.6 million VSL).

A map of the predicted change in $PM_{2.5}$ concentration is shown in Figure S7a. Strong regional trends exist in the change in $PM_{2.5}$ concentration due to the spatial distribution of power plant emissions. In Western states, concentrations generally decrease; Los Angeles County sees the largest decrease in concen-

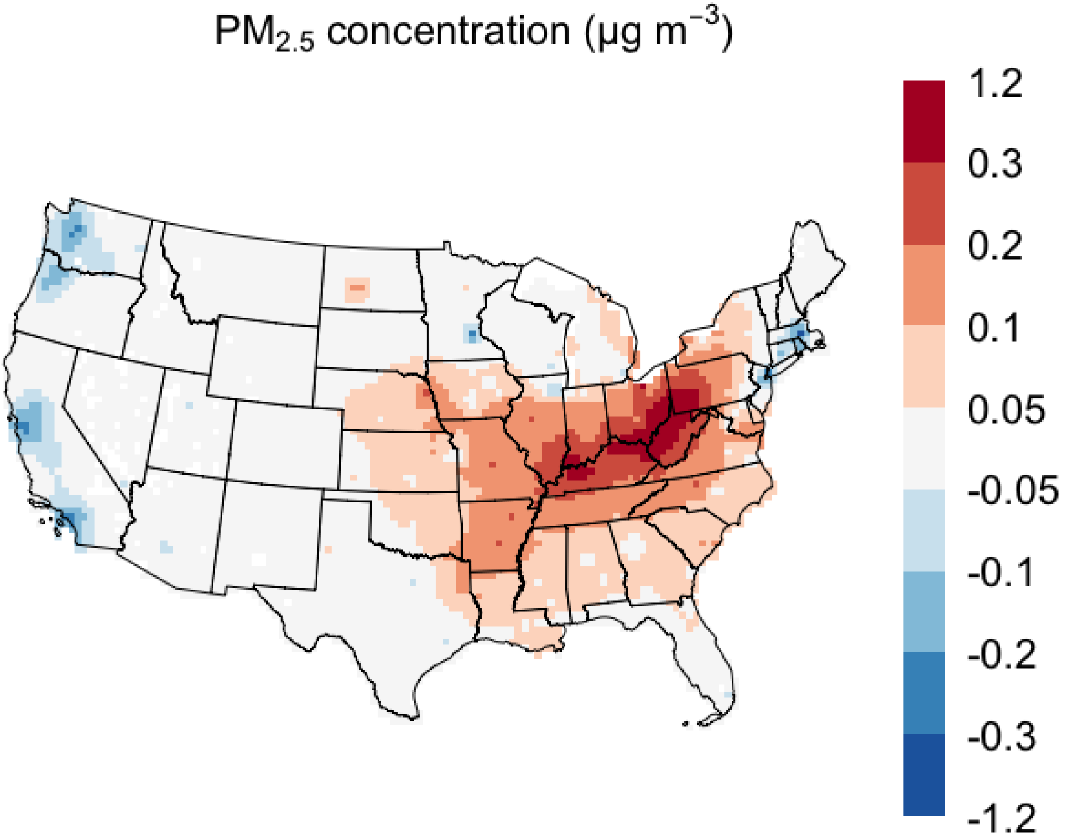

Figure S6: Concentration change estimates across the U.S. using base EASIUR. This model agrees with EASIUR-HR on regional trends in concentration changes.

tration, with a maximum decrease of roughly 1.2 µg/m^3 in the urban core, due to a lack of upwind polluting power plants and significant light-duty passenger vehicle traffic. In Midwest and Eastern states, concentrations largely increase in rural areas, particularly in Minnesota and the Ohio River Valley, while concentrations decrease in urban areas due to the decrease in vehicular emissions. For comparison to other high-resolution models, we process the marginal change in emissions through the original, low-resolution EASIUR model; this plot is shown in Figures S7b. Base EASIUR predicts a population-weighted average decrease in concentration of 0.01 µg/m^3 (social benefit of $800 million).

Figure S8a shows the change in disparity broken down along race/ethnic lines, where change in disparity is measured as the difference between the change in concentration for a specific race group and the change concentration for the population as a whole; negative values indicate that the race group experiences a larger decrease (or smaller increase) in concentration relative to the population as a whole, while positive values indicate that the race group experiences a smaller decrease (or larger increase) in concentration relative to the population as a whole. The two models produce substantially different changes in

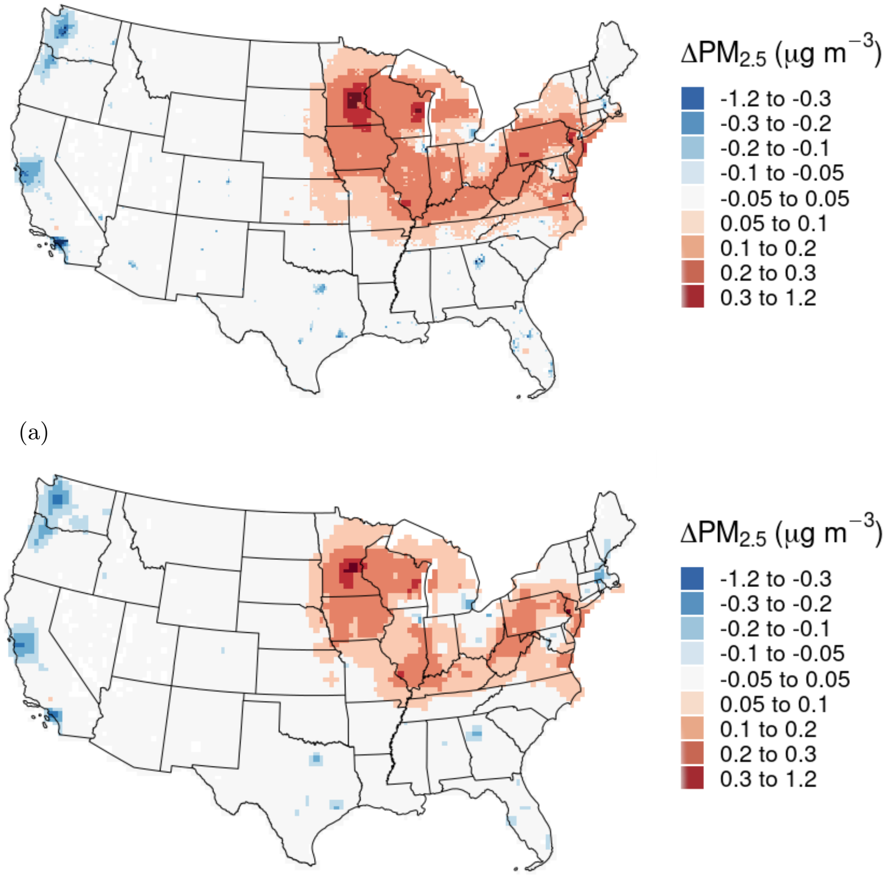

(a)

(b)

Figure S7: Concentration change estimates across the U.S. using two models: (a) high-resolution EASIUR and (b) low-resolution EASIUR. The two models agree on regional trends in concentration changes, with larger increases in concentration across the upper Midwest and in mid-Atlantic states and decreases in concentration across much of the South and the West.

disparity. EASIUR-HR predicts the largest reduction in disparity for Asian communities, with an average reduction of 0.09 $\mu g/m^3$, followed by Hispanic communities with a reduction of 0.08 $\mu g/m^3$, Black communities with a reduction of 0.02 $\mu g/m^3$, and white communities with an increase of 0.02 $\mu g/m^3$.

Figure S8b shows the same change in disparity broken down along income groups for the high-resolution EASIUR model only. There is only a slight trend with income; both high- and low-income groups see reductions in $PM_{2.5}$ concentration, while middle-income groups see a slight increase in concentration. The maximum difference between income groups is just 0.025 $\mu g/m^3$, more than four times smaller than the maximum difference between race/ethnic groups (whites and Asians) of 0.11 $\mu g/m^3$.

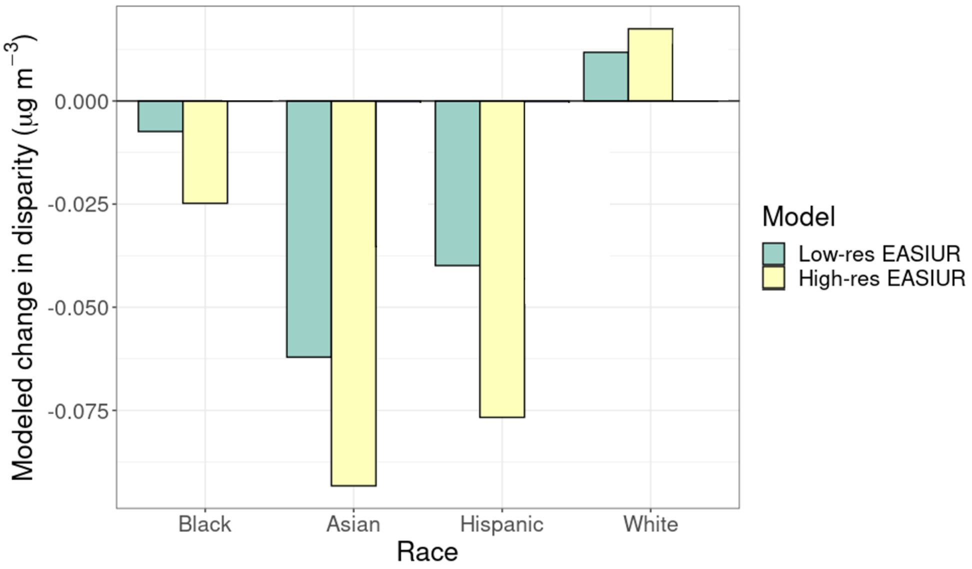

(a)

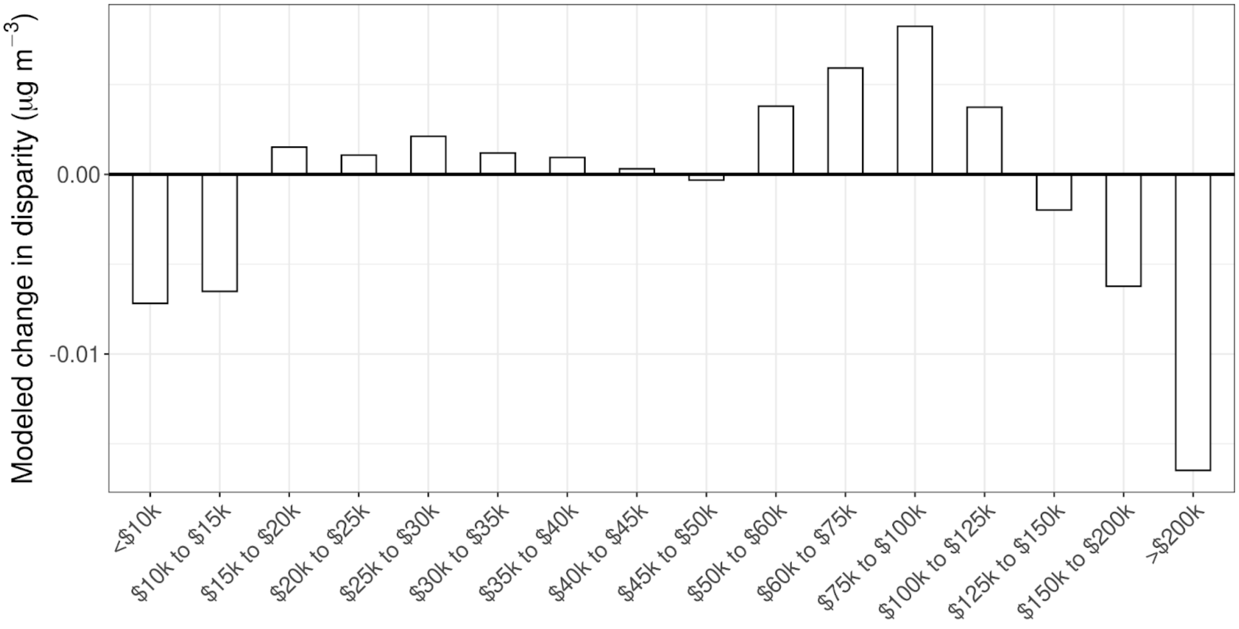

(b)

Figure S8: Comparison of modeled changes in disparities in $PM_{2.5}$ exposure given Schnell et al. vehicle electrification emissions inventory by (a) race, using both EASIUR and EASIUR-HR, and (b) income, using only the high-resolution EASIUR model. EASIUR-HR predicts the largest decreases in disparity for people of color, with an estimated reduction of the disparity between Asians and the population as a whole of 0.09 μg/m^3. Both high- and low-income groups are expected to benefit from vehicle electrification.

4 Model evaluation

We evaluated uncertainty in our model associated with assumptions of constant meteorology over regions of the U.S. This section contains plots of model residuals associated with switching meteorology data. Fig-

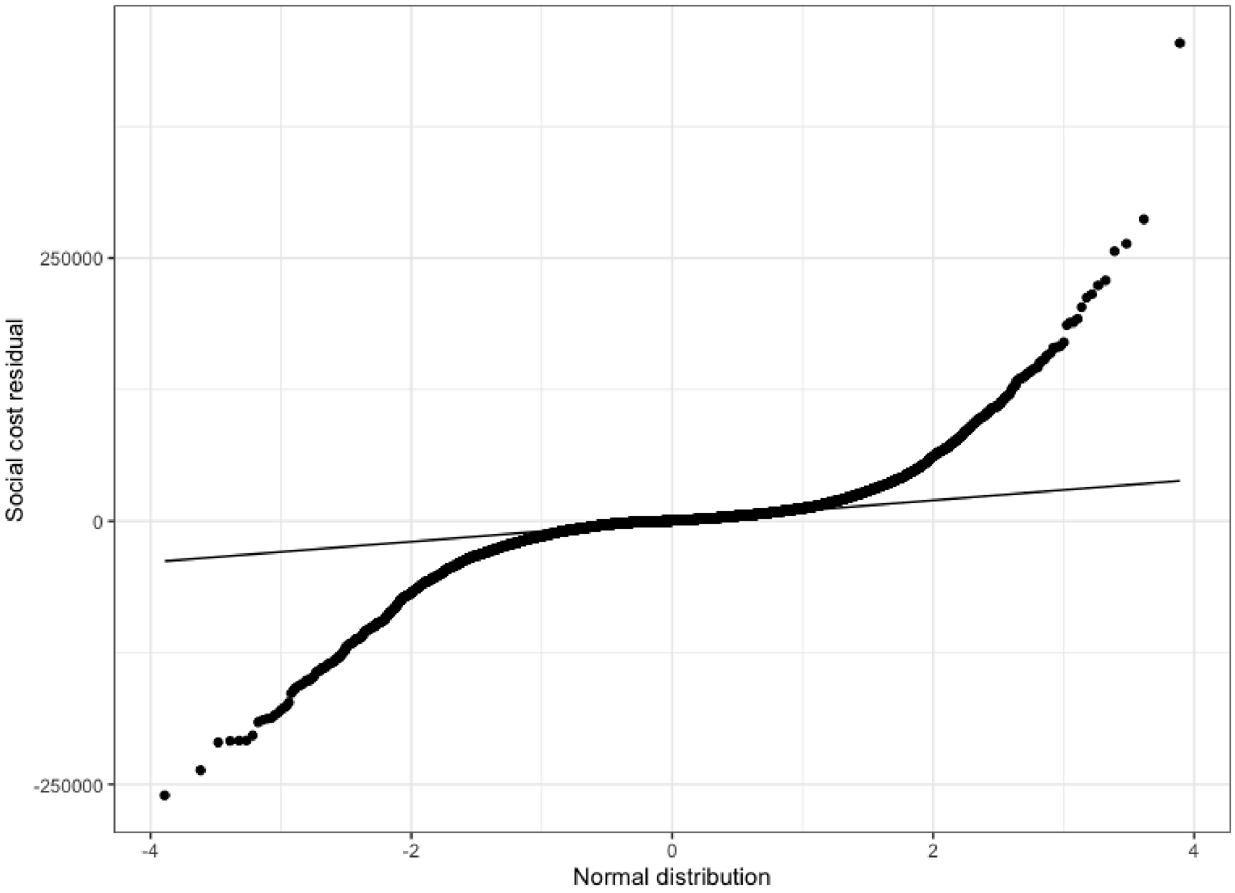

Figure S9: Q-Q plot of social cost residuals.

ure S9 shows a Q-Q plot of the social cost residuals; these have 0 mean, but display wide tails (indicated by deviation from the Q-Q line). Figure S10 shows the same for the disparity metric residuals, which similarly have 0 mean but wider tails than expected for a normal distribution. This indicates our residuals are not normally distributed, but still display a symmetric distribution around 0.

We also plotted our residuals against spatial surrogates included in our assessment of vehicle electrification, shown in Figures S11, S12, S13, and S14. Our residuals have zero conditional mean with spatial surrogates, suggesting that no bias will be introduced into our results as a result of our simplified treatment of meteorology. The residuals do display some heteroskedasticity, with larger variation at smaller values of residuals; this can bias the standard deviations of our results, but will not bias the absolute value of our results.

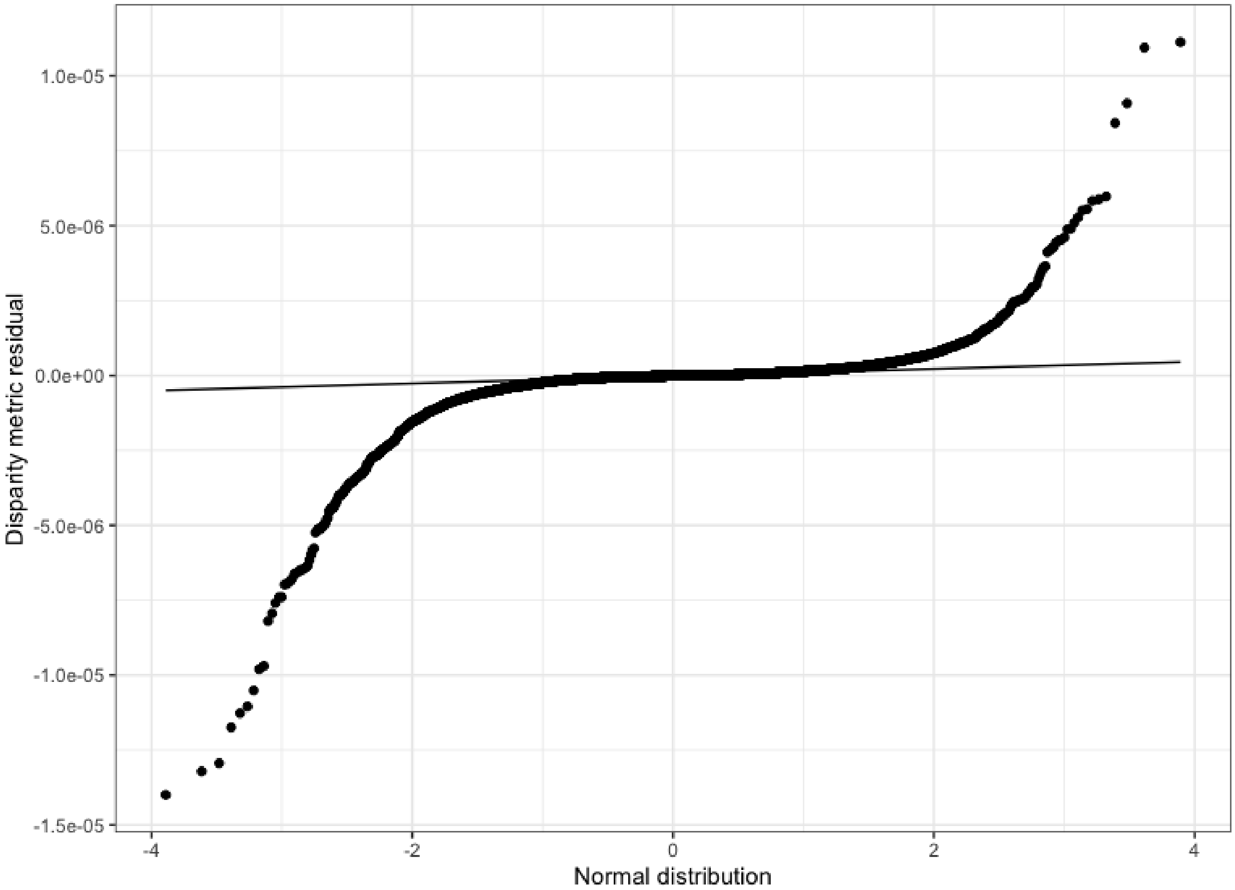

Figure S10: Q-Q plot of disparity metric residuals.

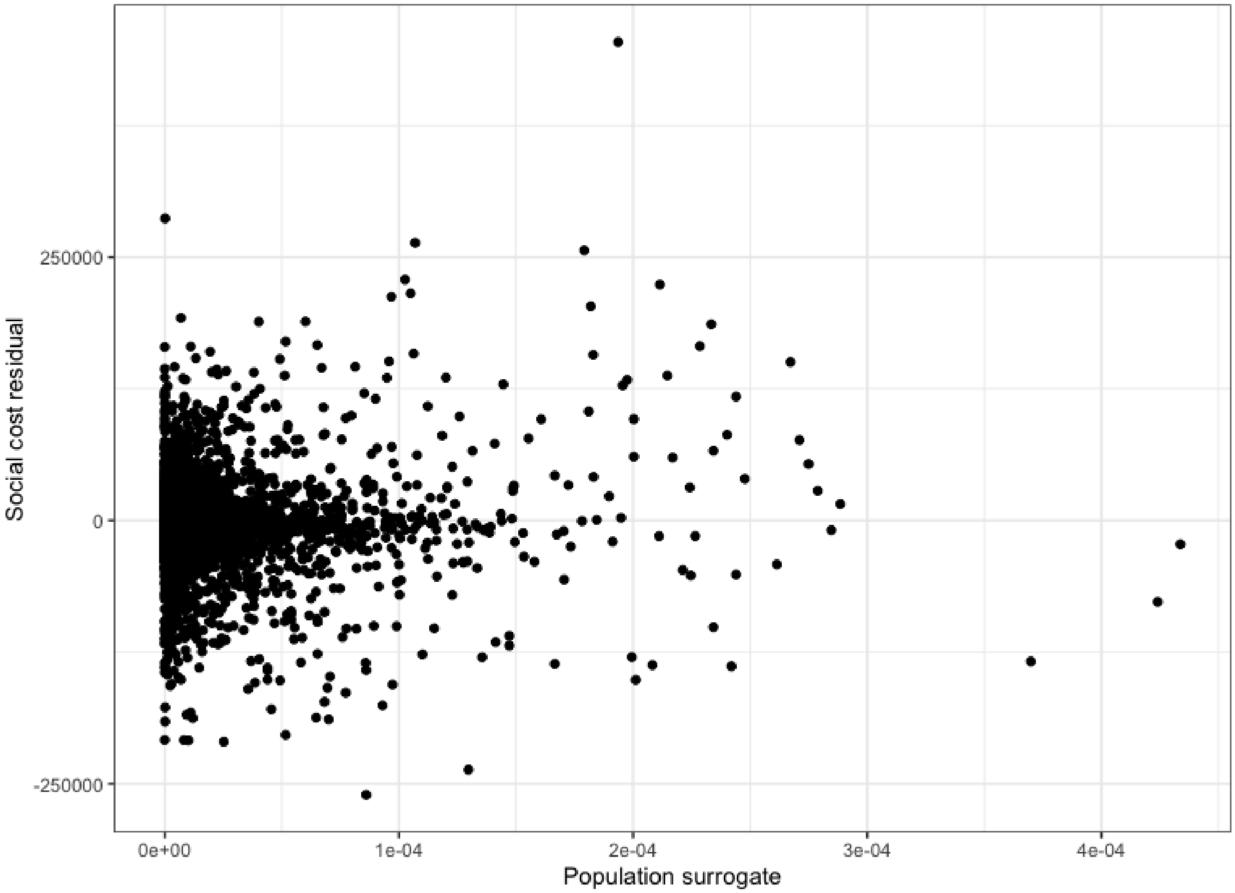

Figure S11: Social cost residuals plotted against population surrogate.

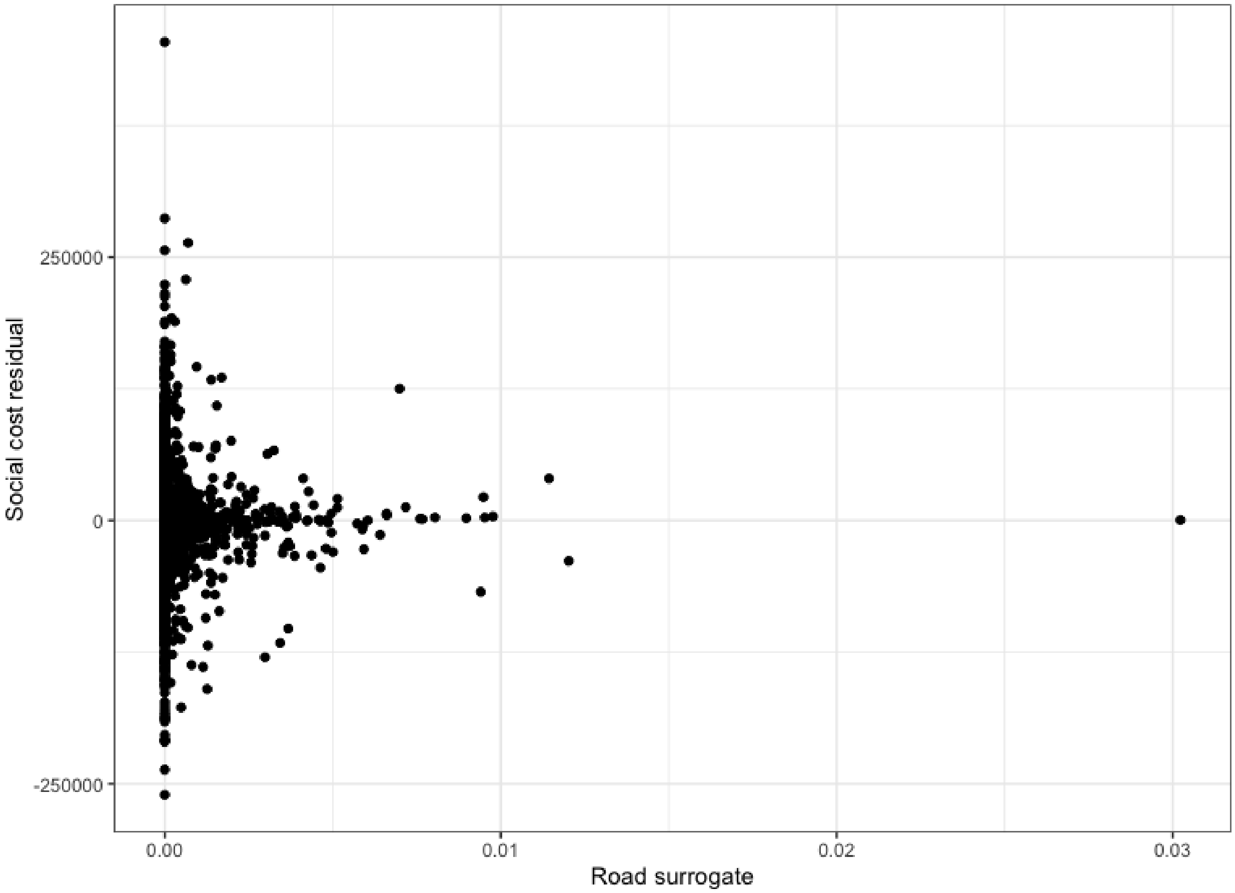

Figure S12: Social cost residuals plotted against road surrogate.

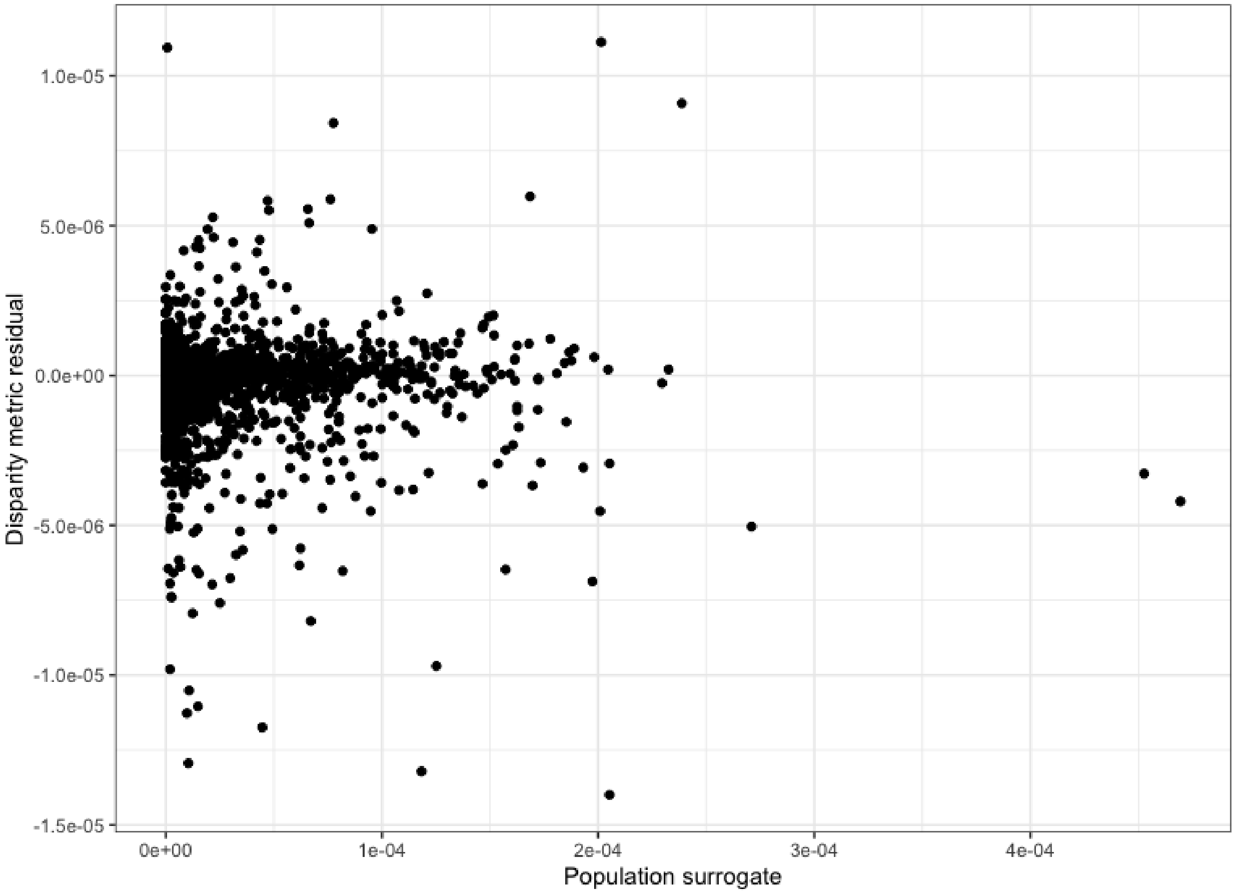

Figure S13: Disparity metric residuals plotted against population surrogate.

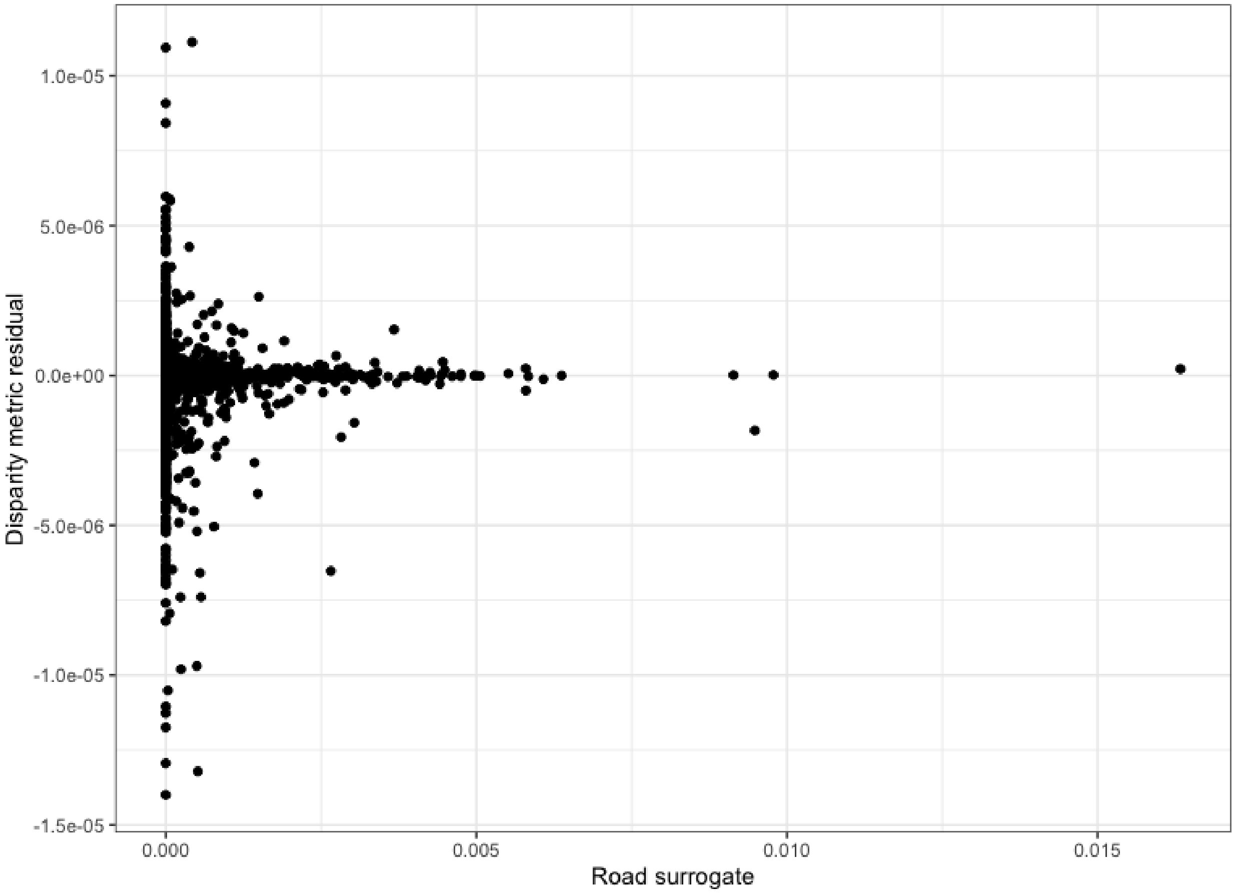

Figure S14: Disparity metric residuals plotted against road surrogate.

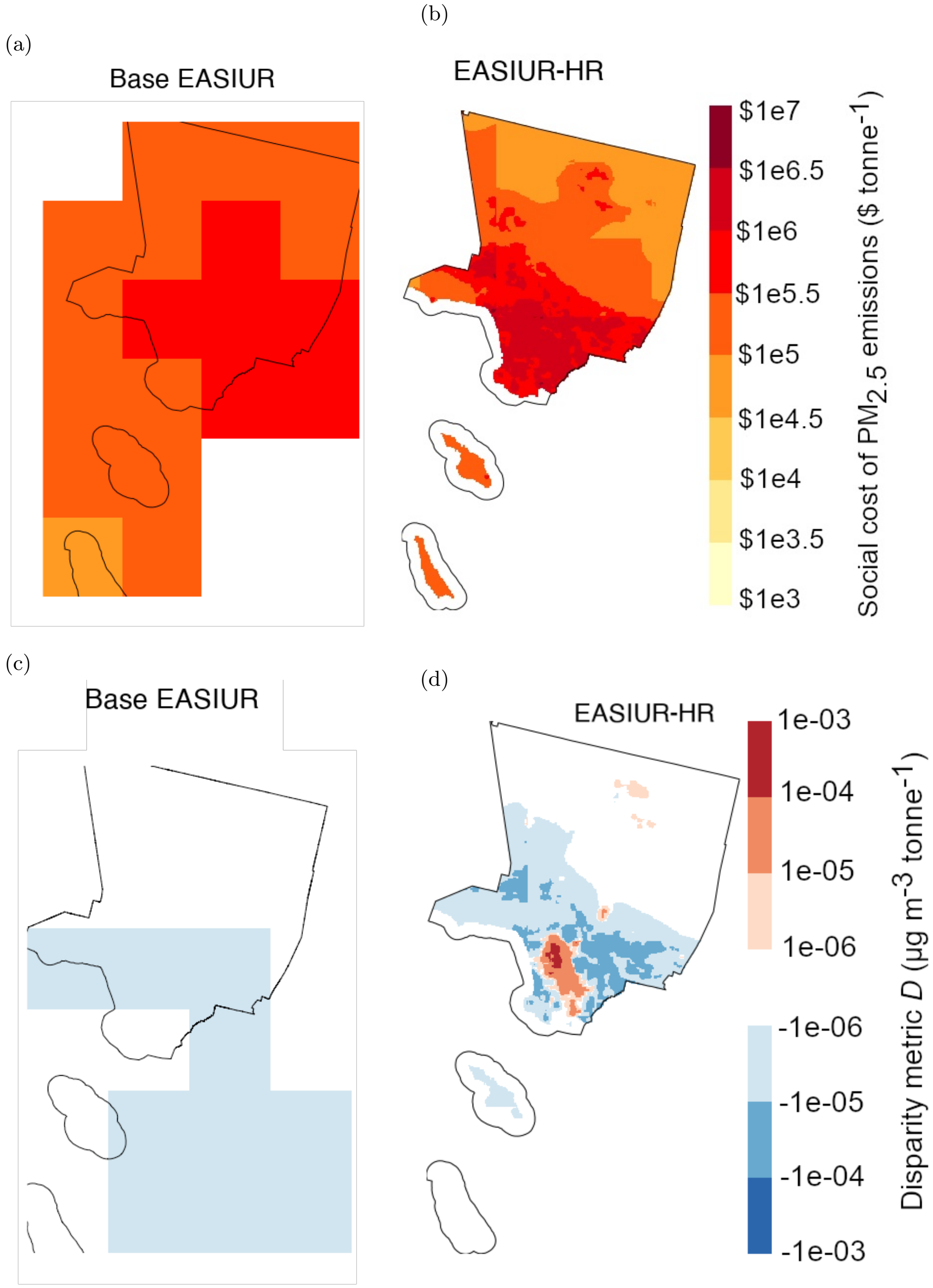

Figure S15: Social costs and disparity metric for Black vs. total population plotted across Los Angeles County using (a, c) base EASIUR and (b, d) EASIUR-HR. Social costs are higher in more densely populated neighborhoods, and the disparity metric shows substantial intra-urban variation depending on local demographics.

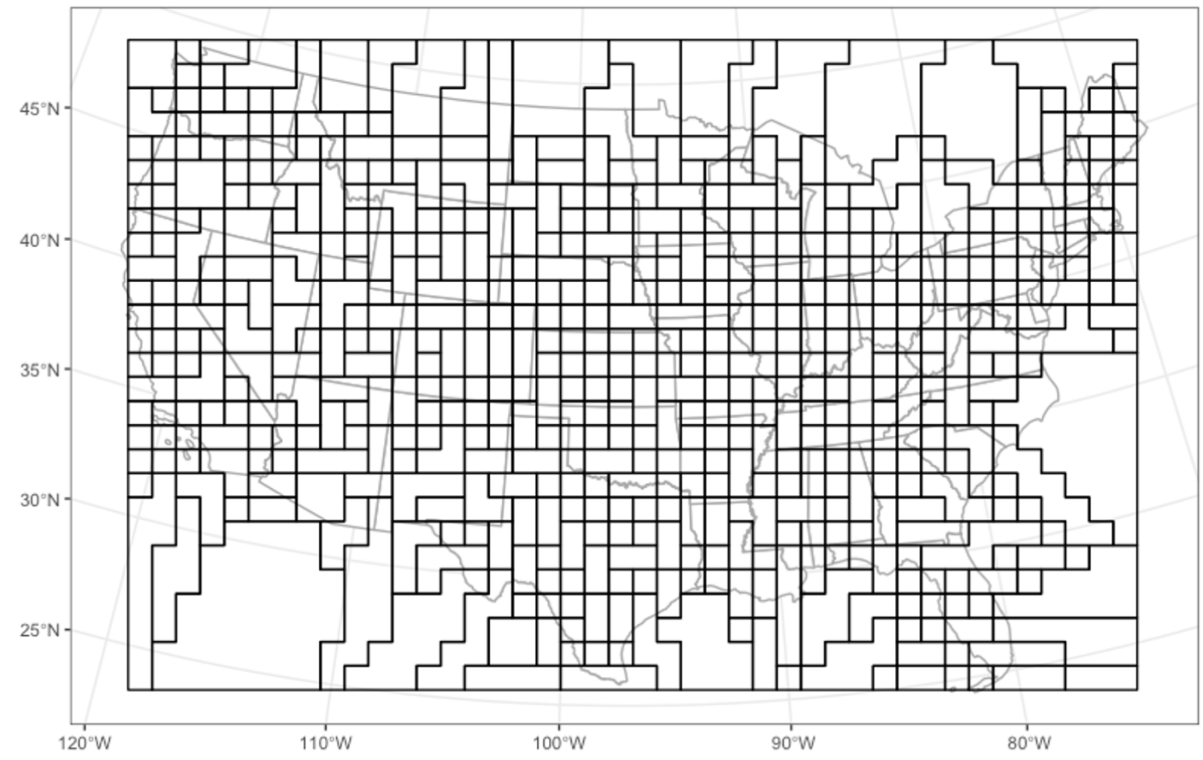

Figure S16: Meteorology regions used in the development of EASIUR-HR. Within each box, the same meteorology data are used.

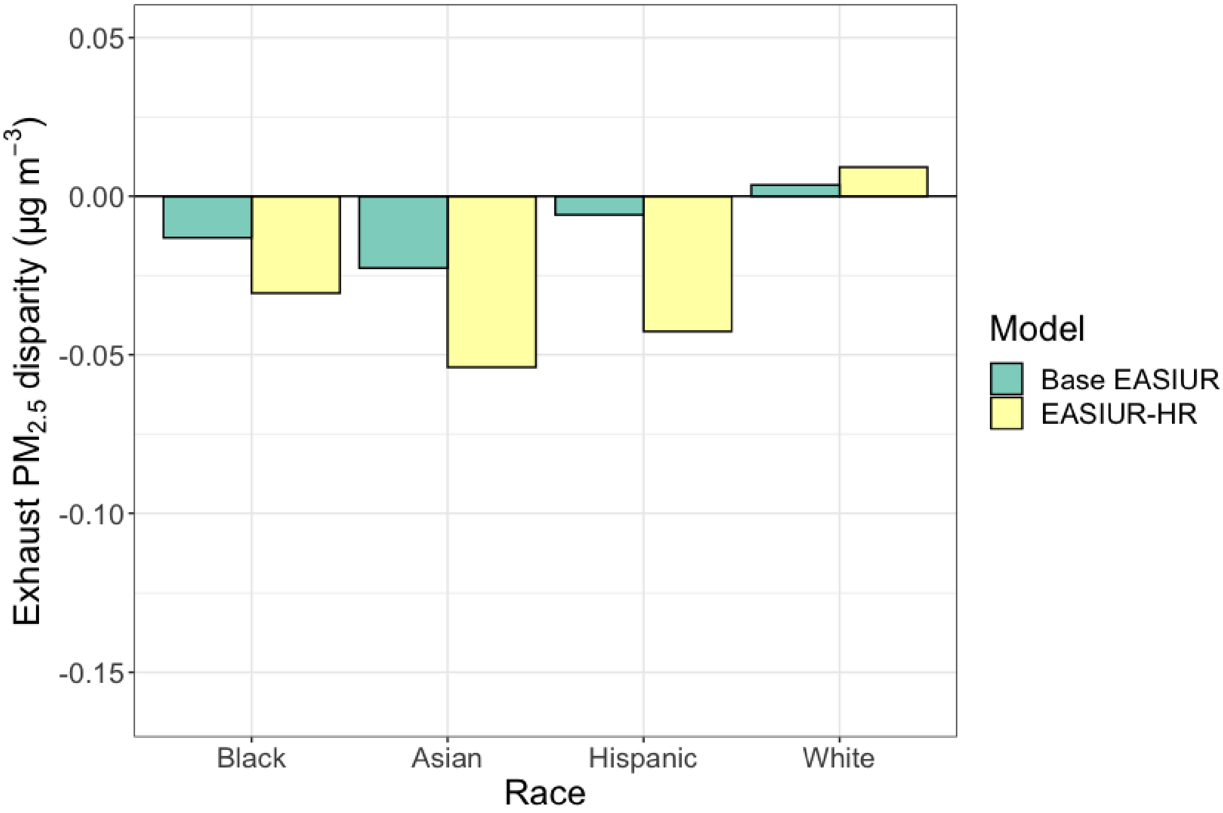

Figure S17: Modeled changes in race/ethnic $PM_{2.5}$ exposure disparity as a result of vehicle electrification by model for vehicle exhaust primary $PM_{2.5}$ emissions only. EASIUR-HR predicts larger changes in disparity than EASIUR for vehicle exhaust $PM_{2.5}$ emissions.

References

(1) Holland, S. P.; Mansur, E. T.; Muller, N. Z.; Yates, A. J. *National Bureau of Economic Research* **2016**.

(2) Schnell, J. L.; Naik, V.; Horowitz, L. W.; Paulot, F.; Ginoux, P.; Zhao, M.; Horton, D. E. *Atmospheric Environment* **2019**, *208*, 95–102.
